# CD19嵌合抗原受体T细胞治疗弥漫大B细胞淋巴瘤的复发机制及应对策略

**DOI:** 10.3760/cma.j.issn.0253-2727.2022.09.014

**Published:** 2022-09

**Authors:** 琪 张, 毅 肖

**Affiliations:** 华中科技大学同济医学院附属同济医院血液内科，武汉 430030 Department of Hematology, Tongji Hospital, Tongji Medical College, Huazhong University of Science and Technology, Wuhan 430030, China

嵌合抗原受体T细胞（CAR-T）免疫疗法是治疗恶性肿瘤的新兴疗法，尤其在血液肿瘤的治疗中展示出显著疗效。CD19是一种在B细胞成熟的各个阶段均表达的细胞表面蛋白，均匀表达于95％以上的B细胞恶性肿瘤表面，被认为是治疗B细胞恶性肿瘤的理想靶点。对于复发/难治B细胞非霍奇金淋巴瘤（NHL）患者，CD19 CAR-T治疗后总反应率为52％～82％，40％～54％的患者可以达到长期完全持续的缓解[1]。CAR-T疗法虽然给复发/难治弥漫大B细胞淋巴瘤（R/R DLBCL）患者带来了福音，但是仍有部分患者发生复发，导致结局不佳。本文综述了可能复发的原因，并提出相应的应对策略，以便为提高CAR-T疗效提供有效参考。

一、CAR-T治疗后复发机制

（一）患者本身因素

1. 疾病状态：诸如身体不良状态、桥接治疗要求和基线LDH升高等因素与CAR-T疗效不佳相关，研究发现LDH升高、ECOG≥2分及伴重要脏器并发症的患者CAR-T治疗后无进展生存（PFS）和总生存（OS）率显著降低[2]。疾病复发的独立预测因素包括C反应蛋白（CRP）升高、结外累及部位≥2个、总代谢肿瘤体积（TMTV）>80 ml[3]，同时出现结外部位累及≥2个和TMTV>80 ml的患者预后最差。以上因素在不同程度上影响CAR-T治疗效果。

2. 抗原逃逸：尽管抗CD19 CAR-T细胞治疗对R/R DLBCL显示出强大的抗肿瘤活性，但高达25％的患者由于抗原丢失或下调而预后不佳[4]。靶缺失是一种特征明确的耐药机制，通过选择具有CD19外显子剪接或基因突变的肿瘤细胞而导致CD19表达的缺失/改变。对复发患者进行RNA测序发现存在CD19的替代剪接，类似于B-ALL患者中外显子2和（或）外显子5/6缺失的情况以及其他几种新的剪接接头，CD19外显子2发生从头移码和错义突变，同时存在CD19的备选外显子2剪接[5]，由于CD19表位因剪接/突变机制丢失，B细胞恶性肿瘤中无法表达CD19，使其不能被CD19 CAR-T细胞识别而逃避肿瘤杀伤效应。肿瘤抗原逃逸已成为疾病控制的长期挑战，需要靶向其他靶点的CAR-T细胞补救治疗，未来的CAR和其他基于抗体的治疗手段应考虑设计为靶向必需外显子，以防止抗原逃逸。

3. 肿瘤因素：导致CAR-T治疗失败的肿瘤因素可能是肿瘤体积过大或肿瘤微环境中T细胞抑制配体（如PD-L1）的表达。高肿瘤体积与DLBCL较低的持久反应相关，肿瘤负荷高的患者外周血中炎症相关分子分泌会增加，如铁蛋白、IL-6、IL-15、TNF-α及高Ang2/Ang1比率，高肿瘤负荷还与肿瘤中较高水平的IFN信号相关，分析显示肿瘤IFN和巨噬细胞基因特征在早期复发的患者中富集[6]。肿瘤微环境中存在免疫抑制细胞和细胞因子等多种抑制分子，自身免疫抑制可能会抑制CAR-T细胞的活性，通过免疫检查点分子和配体之间的相互作用，抑制信号可以逃避免疫监视，导致T细胞衰竭和肿瘤耐受。CAR-T细胞中PD-1表达的显著上调降低了抗肿瘤免疫反应水平，并促使肿瘤细胞逃离免疫系统。

4. 患者本身T细胞质量：CAR-T细胞疗法通常用于接受多次强化化疗的患者，前期化疗次数过多会损害T细胞的功能和质量，且多数患者曾接受具有细胞毒性反应的治疗，导致无法收集到治疗所需的足够数量的T细胞或收集到功能受损、效力和抗肿瘤活性方面功能欠佳的T细胞。此外，即使初治肿瘤患者也可能存在T细胞功能障碍，与来自健康供者的T细胞相比，DLBCL患者来源的T细胞在多克隆激活时增殖较少[7]。T细胞功能障碍是B细胞淋巴瘤的明确危险因素，其制备的产品质量也会下降，失败或复发的风险更高。因此应考虑CAR-T线数提前，避免多次化疗造成T细胞损伤及减少，高危患者的早期单采最好在挽救性化疗之前，以改善CAR-T细胞的质量和随后的治疗结果。

（二）CAR-T因素

1. CAR-T结构：CAR-T分子结构对于产品疗效和持久性有较大影响。研究表明分泌IL-2的CAR-T细胞更具效力和持久性，能够克服T细胞介导的抑制作用，Kagoya等[8]开发了一种将IL-2信号传导的下游成分整合到CAR-T细胞中的构建体，与目前CAR-T细胞相比，显示出优异的疗效和持久性。另外，仅改变CAR的铰链和跨膜区域即可对CAR-T细胞产生细胞因子有重要影响[9]，ZUMA-1、Juliet和Transcend研究中的CD19 CAR-T细胞具有相似的结构，含有相同的单链可变片段（FMC63），并使用CD3作为细胞内信号，但3种产品跨膜域和共刺激分子组合不同，便出现疗效差异[10]。表达具有来自CD28的铰链和跨膜结构域的抗CD19 CAR-T细胞比表达除具有CD8α衍生铰链和跨膜结构域外相同的CAR-T细胞产生炎性细胞因子的水平更高[9]。

2. T细胞耗竭：多种因素影响CAR-T细胞的活性和持久性，如T细胞亚型、衰竭以及与肿瘤微环境的相互作用。T细胞耗竭是指反复抗原暴露导致T细胞功能丧失，因为存在共刺激分子，即使没有持续的抗原暴露，CAR-T细胞也不可避免会被消耗[10]，引起效应细胞功能削弱。T细胞耗竭一般与持续的抗原刺激、CAR结构的共刺激域、效应器功能丧失、免疫抑制因子和免疫调节细胞、多种抑制性受体共表达产生的负调控有关，包括PD-1、CTLA-4、T细胞免疫球蛋白和含黏蛋白结构域-3（Tim-3）、淋巴细胞活化基因-3（Lag-3）、T细胞免疫球蛋白和ITIM结构域（TIGIT）等[11]。

二、应对策略

（一）增强CAR-T功能

提高CAR-T疗效的策略包括：（1）优化CAR结构域之间的间隔区长度[12]；（2）合并额外的共刺激结构域（“第三代”CAR）[13]；（3）包含细胞因子表达结构的CAR-T。

CAR-T细胞具有使其能够扩增的额外的激活和识别域，第一代CAR-T细胞增殖和存活能力弱且抗肿瘤活性有限，加入共刺激域的第二代CAR-T细胞扩增能力、持续性和信号强度得以提高，而将共刺激域整合到CAR中的“第三代”（3G）CAR-T细胞可以显著改善CAR-T细胞的增殖和体内持久性[13]。最佳共刺激域中CD28和4-1BB在CAR-T临床试验应用最广泛，CD28 CAR结构显示出更高的峰值扩增，基于4-1BB的结构则显示出更长的持续性[14]。第四代CAR-T细胞通过分泌IL-12、IL-15、IL-18等免疫调节性细胞因子激活对肿瘤细胞的杀伤作用，显示出更强的抗肿瘤疗效，并可以存活于局部肿瘤免疫抑制微环境中，设计能够通过释放细胞因子激活CAR-T细胞的人工抗原呈递细胞能够刺激和扩增CAR-T细胞的数量[15]。

（二）换用人源化或全人源化CAR-T

免疫介导的CAR-T细胞排斥反应可通过用免疫原性较低的人源化或全人源化CAR-T细胞来解决。由于鼠源CAR序列的免疫原性导致CAR-T细胞无法活化及持续存在，用人源化scFv替换鼠源scFv具有很强的持久性和抗肿瘤作用，可减少人体对CAR序列产生的免疫应答，减少宿主免疫系统对CAR-T细胞的破坏，使CAR-T细胞得以扩增和持久作用。源自鼠的scFv的框架区并与CD8α跨膜结构域、4-1BB共刺激结构域、CD3ζ信号域结合的新型人源化抗CD19 CAR-T细胞（h1CAR19-8BBζ）具有强大的抗肿瘤活性且产生细胞因子较少[16]。全人源化CAR-T可能消除鼠源免疫排斥反应，增加抗CD19 CAR-T细胞的持久性，包含全人源化ScFv的第二代全人源化抗CD19 CAR-T可有效破坏CD19^+^肿瘤细胞，并以CD19特异性的方式释放Th1细胞因子，包括IL-2、IFN-γ和TNF-α[17]。与鼠CD19 CAR-T细胞相比，人源化CD19 CAR-T细胞的转导增殖效率更高，在脑脊液、外周血和骨髓中的存活时间更长，在体外和小鼠中对肿瘤细胞均表现出更好的效果。

（三）增强肿瘤抗原表达

用于淋巴瘤的CD19 CAR-T细胞疗法取决于与肿瘤细胞表面CD19靶标的结合。抑制性信号如PD-L1可能会减少表位扩散，并因肿瘤抗原改变而增加失败风险。克服CAR靶向肿瘤逃逸的一个方法是增加靶细胞上抗原的再表达或增强诱导新的天然抗肿瘤免疫应答。临床前数据表明，由于炎症过程增加，CAR-T细胞会增加肿瘤新抗原的表达，被宿主免疫细胞识别后能运输天然免疫细胞，并通过靶向表位以外的其他表位攻击抗原阴性肿瘤细胞[18]。经抗CD20单克隆抗体利妥昔单抗治疗后，给予抗甲基化药物阿扎胞苷可在淋巴瘤细胞上重新诱导CD20表达。总之，组蛋白去乙酰化酶抑制剂和阿扎胞苷等增加抗原在靶细胞上再表达可以增强CAR-T细胞的抗原识别和抗肿瘤活性。

（四）多靶点CAR-T联合

使用靶向一种以上肿瘤抗原的CAR-T细胞有助于降低由抗原逃逸和谱系转换引起的复发风险，还可能减少肿瘤抗原异质性的影响，例如CD19和CD123或更常见的CD19和CD22。联合应用抗CD19和CD20 CAR-T细胞治疗DLBCL可行且毒性可控[4]。同时靶向CD19和CD22的CAR-T细胞治疗可能减少肿瘤逃逸，并增加对肿瘤细胞的杀伤作用[19]，比靶向单个CD19或CD22抗原获得更持久的缓解。通过将衍生自FMC63单克隆抗体的CD19 scFv或衍生自Leu-16单克隆抗体的CD20 scFv与CD8的铰链和跨膜区以及4-1BB和CD3 ζ的胞质区结构连接成一系列串联CARs（TanCARs），发现TanCAR7 T细胞表现出CD19和CD20的双重抗原靶向性并具有与其强大的抗肿瘤活性有关的稳定免疫突触（IS）结构[20]，在体内诱发有效且持久的抗肿瘤反应，试验证明了TanCAR的安全性、有效性、低毒性以及潜在减少R/R NHL患者抗原逃逸复发的能力。考虑到两种CAR-T细胞产物同时使用时可能会不同程度地扩增，并有丢失一个CAR-T细胞产物的风险，双特异性CAR-T细胞基因结构更紧凑，有利于载体包装和提高转导效率，当仅与一种靶抗原结合时，双特异性CAR-T细胞能被有效激活，两种抗原同时丢失的可能性也很低，可有效防止丢失其中一种抗原导致的复发，增强CAR-T细胞治疗的长期疗效[21]。

（五）解除免疫抑制机制

肿瘤微环境中PD-1的上调抑制CAR-T细胞功能，可见增强CAR-T功能、减轻免疫抑制的一种方法是直接破坏PD-L1/PD-1免疫抑制轴，阻断PD-1/PD-L1通路不仅可以增加T细胞的数量，改善CAR-T细胞的功能，还可以通过改变肿瘤抑制微环境来增强T细胞的抗肿瘤作用。另一种方法是通过基因工程结合CAR-T细胞治疗和免疫抑制剂，表达PD-1抗体单链可变片段的T细胞可以阻断免疫细胞PD-1和肿瘤细胞PD-L1之间的相互作用，解除免疫抑制[22]，利用基因工程将PD-1基因敲除或将PD-1抗体“移植”入CAR-T细胞是一个研究的新方向。

CAR-T细胞治疗联合PD-1抑制剂或细胞因子抑制剂可以改善整体或局部免疫环境，在血液肿瘤的治疗中显著增强抗肿瘤效果。一例接受CD19 CAR-T细胞治疗出现难治性DLBCL的患者，采用PD-1阻断治疗后CAR-T细胞明显扩增，抗肿瘤反应强烈，肿瘤负荷减轻[23]。一例滤泡淋巴瘤患者CAR-T细胞治疗效果不佳，接受低剂量PD-1阻断后病情持续缓解10个月以上[24]。ZUMA-6试验中axi-cel联合阿替唑珠单抗治疗难治性DLBCL的结果显示，10例可评估患者中总有效率为90％，完全缓解率为60％[25]，具有合理的安全性。除PD-1/PD-L1途径外，CTLA-4/B7-1相互作用同样抑制T细胞应答，阻断其他免疫检查点是否也能提高CAR-T细胞疗效尚待进一步研究。

（六）换用不同嵌合抗原

一些早期临床试验表明，针对淋巴瘤中的其他抗原（如CD30、43κ轻链和CD20）也具有适度的临床疗效和安全性[26]，这提高了构建针对多个抗原表位的CARs或使用不同靶点（同时或顺序）给药多种CARs的可能性。CD22在大多数B细胞恶性肿瘤（如DLBCL和其他B-NHL）的肿瘤细胞表面表达，可用于抗CD19 CAR-T细胞治疗后失败或再复发的患者，有研究报告抗CD22 CAR-T细胞治疗在此类患者中诱导出高应答率[27]。CD70在DLBCL等多种恶性肿瘤中均有表达，高水平的CD70表达与DLBCL的不良预后相关，提示该分子可能成为DLBCL的潜在治疗靶点[28]。

（七）联合体系治疗

1. 放疗联合CAR-T细胞治疗：在胰腺癌模型中发现，暴露于低剂量照射的肿瘤对CAR-T细胞（其中包括缺乏CAR靶点的肿瘤细胞）更加敏感，这有利于减少抗原阴性肿瘤的复发。放疗诱导肿瘤内T细胞杀伤效应增强可能是由肿瘤细胞表面MHC类分子表达的增加、伴随的抗原呈递改善以及细胞毒性T淋巴细胞的增强识别介导的，也可能是与树突细胞的激活、新抗原呈递的改善和MHC类分子的增加相关[29]。全身和局部低剂量放疗都能使肿瘤对CAR-T细胞的杀伤作用更加敏感，放疗联合CAR-T细胞治疗可能比单纯CAR-T细胞治疗更有效。

2. 自体造血干细胞移植（ASCT）序贯CAR-T细胞治疗：我们中心的研究显示，ASCT序贯CAR-T可以使患者获益，还可使存在中枢侵犯或原发中枢神经系统淋巴瘤（CNSL）的患者获得持续缓解。42例患者接受大剂量化疗桥接ASCT（HDT-ASCT）序贯CD19/22 CAR-T细胞输注，总有效率为90.5％（95％*CI* 77.4％～97.3％），2年PFS率为83.3％（95％ *CI* 68.2％～91.7％），且细胞因子释放综合征（CRS）和神经毒性均可逆，这些数据说明HDT-ASCT+CD19/CD22 CAR-T细胞鸡尾酒疗法可以显著改善对挽救性化疗不敏感或无反应的R/R侵袭性B-NHL患者的不良预后，耐受性良好且毒性可控，具有优越的抗淋巴瘤疗效[30]。13例CNSL患者（4例PCNSL和9例SCNSL）在ASCT后顺序输注CD19/22 CAR-T细胞，总有效率和完全缓解率分别为81.81％和54.55％，估计1年PFS和OS率分别为74.59％和82.50％，CNSL患者可获得长期缓解且无严重毒性，两例CR患者在我们的试验中均获得长期无疾病/无复发生存，表明ASCT序贯CAR-T细胞输注可对R/R高危CNSL患者产生长期巩固效应[31]。需要说明的是，在ASCT序贯CAR-T细胞免疫治疗之前应给予合适的预处理方案，例如对疾病稳定或疾病进展患者给予大剂量化疗以获得完全或部分缓解，最大程度杀死肿瘤细胞、缩小肿瘤体积并破坏免疫抑制微环境，从而增强CAR-T细胞的功能和持久性，以提高治疗效果。

（八）通用型CAR-T

如患者本身T细胞数量不足或者功能较差即建议采用通用型CAR-T。通用型CAR-T细胞是一种来自同种异基因健康供者的肿瘤抗原特异性T细胞，通过基因编辑破坏TCR基因和（或）HLA-I类基因座来有效减少移植物抗宿主病（GVHD）发生[32]。PBCAR0191是一种通用型同种异基因CD19靶向CAR-T细胞疗法，由来源于合格供者的T细胞经过基因编辑去除内源性TCR的表达，并将CAR插入同一位点制备而成，在治疗人类白细胞抗原（HLA）不匹配、肿瘤表达CD19的B细胞恶性肿瘤患者时可达到抗肿瘤的效果，同时减少GVHD发生，初步数据表明其不良反应多数为轻度，未发生严重CRS/免疫效应细胞相关神经毒性综合征[33]。ALLO-501A是一种不含利妥昔单抗识别结构域的抗CD19同种异基因CAR-T，利用基因编辑破坏TCRα基因后有可能降低GVHD风险，同时编辑CD52基因可能允许使用ALLO-647（一种人源化抗CD52 mAb）选择性耗竭宿主T细胞，ALPHA2研究结果表明ALLO-501A在CAR-T初治患者中的总有效率为56％，其中完全缓解率为44％[34]。通用型CAR-T具有适用更多患者、制备方便、不受患者自身T细胞影响等优势，有望提高现有CAR-T疗效。

三、小结

综上所述，CAR-T免疫疗法的应用在血液肿瘤及其他实体瘤的应用中有明显疗效，我们需要深入研究复发机制及复发后如何进行CAR-T挽救性治疗，在达到提高疗效的同时减少复发率，让这种极具前景的疗法发挥其最大的效用。
